# The Role of Extracellular Vesicles as Diagnostic Tools in Gut-Brain Axis Disorders

**DOI:** 10.1007/s12035-025-05645-3

**Published:** 2026-01-07

**Authors:** Patricia Marçal da Costa, Paulo Iury Gomes Nunes, Gabriella Cunha Vieira Ciurleo, José Wagner Leonel Tavares Junior, Pedro Braga Neto, Ludmila Belayev, Reinaldo Barreto Oriá

**Affiliations:** 1 Laboratory of the Biology of Tissue Healing, Ontogeny and Nutrition, Department of Morphology and Institute of Biomedicine, School of Medicine, Federal University of Ceará, Fortaleza, Brazil; 2 Department of Clinical Medicine, Faculty of Medicine, Neurology Section, Federal University of Ceará (UFC), Fortaleza, CE Brazil; 3 Center of Health Sciences, State University of Ceará (UECE), Fortaleza, Ceará Brazil; 4 School of Medicine, Neuroscience Center of Excellence, Louisiana State University Health Sciences Center, New Orleans, LA USA

**Keywords:** Biomarkers, Diagnostics, Extracellular vesicles, Gut–brain axis, Isolation methods, Precision medicine

## Abstract

The gut–brain axis represents a dynamic two-way signaling network whose dysregulation has been implicated in a wide range of neurogastrointestinal disorders. In this context, extracellular vesicles (EVs) have emerged as critical mediators of intercellular signaling and as promising non-invasive biomarkers. Derived from host and microbial cells, EVs carry bioactive cargo—including proteins, lipids, nucleic acids, and metabolites—that reflect the physiological or pathological state of their cells of origin. Their ability to cross biological barriers, such as the blood–brain barrier, underscores their potential for diagnosing and monitoring gut–brain axis dysfunctions. In this mini-review, we integrate microbial and brain-derived EVs within the framework of gut–brain axis disorders and propose three translational “diagnostic niches”: microbial EVs as systemic markers of dysbiosis and immune activation, brain-derived EVs as liquid biopsies of the central nervous system (CNS) pathology, and engineered or technologically captured EVs as platforms for point-of-care testing. We summarize recent mechanistic insights, highlight disease-specific evidence in irritable bowel syndrome, inflammatory bowel disease, neurodegenerative, and psychiatric conditions, and critically appraise emerging isolation and analytical technologies in light of MISEV2023 recommendations. Finally, we discuss current limitations and translational hurdles, outlining how standardized EV-based diagnostics may be incorporated into precision medicine strategies targeting neurogastrointestinal diseases.

## Introduction

The gut–brain axis constitutes a dynamic bidirectional communication system that integrates neural, endocrine, immune and metabolic signals between the gastrointestinal tract and the central nervous system (CNS). This coordinated network is essential for maintaining homeostasis and regulating complex functions such as appetite, mood, immune responses and the integrity of biological barriers, including the intestinal mucosa and the blood–brain barrier (BBB) [1–3]. Within this intersystemic framework, the gut microbiota emerges as a central regulatory agent, capable of profoundly influencing neurobehavioral circuits, while the brain, in turn, modulates intestinal motility, secretion and permeability [2–4].

In recent years, extracellular vesicles (EVs) have been recognized as key mediators at this interface, acting as carriers of lipids, proteins, metabolites and nucleic acids that reflect the functional and pathological state of their cells of origin [5–7]. Secreted by multiple cell types—including neurons, astrocytes, intestinal epithelial cells and components of the microbiota—these nanoparticles actively participate in intercellular signaling, regulation of redox balance, clearance of aggregated proteins and modulation of immune responses in both peripheral and central tissues [8–11]. Their activity can be beneficial, promoting the maintenance of cellular integrity, or deleterious, by transporting inflammatory mediators and propagating pathological alterations under conditions such as dysbiosis or neurodegeneration [6, 12–14].

In diseases such as irritable bowel syndrome (IBS), Alzheimer’s disease (AD) and intestinal dysbiosis associated with metabolic syndrome, EVs have proven to be not only promising biomarkers but also potential pathogenic modulators. Owing to their ability to cross biological barriers, including the BBB, EVs provide a non-invasive route to access both the cerebral and intestinal microenvironments, enabling the early detection of molecular targets associated with gut–brain axis disorders [15–17].

The consolidation of EVs as diagnostic and therapeutic tools has been driven by advances in isolation, purification and characterization technologies, including high-sensitivity microfluidics, mass spectrometry, nanoparticle tracking analysis and integrated multi-omics platforms [17–19]. These approaches have enhanced the detection of specific EV subpopulations, such as those derived from neurons, glial cells and gut bacteria, thereby increasing accuracy in patient stratification and monitoring individualized therapeutic responses [20–22].

In light of emerging evidence, this mini-review aims to provide an integrative and translational overview of the role of EVs within the context of the gut–brain axis. We focus on how EVs derived from the CNS and gut microbiota converge to shape neurogastrointestinal disorders and organize current data into three diagnostic niches: (1) microbial EVs as systemic markers of dysbiosis and immune activation, (2) brain-derived EVs (BDEVs) as CNS liquid biopsies in gut–brain-related diseases, and (3) engineered or technologically captured EVs as platforms for point-of-care (POC) applications. Furthermore, we highlight emerging methods for EV isolation and analysis, discuss how current studies align (or not) with MISEV2023 guidelines and identify major gaps and translational hurdles that must be addressed for EV-based diagnostics to enter clinical practice.

## Extracellular Vesicles

EVs are membrane-bound nanoparticles actively released by cells under both physiological and pathological conditions. These particles typically range from ~ 30 nm to more than 1 µm and encompass exosomes, microvesicles and apoptotic bodies, which partially overlap in size and composition [14, 17]. EVs transport proteins, lipids, nucleic acids and metabolites, thereby modulating cellular functions at a distance and participating in processes such as immune regulation, protection against oxidative stress and maintenance of barrier integrity [5, 6, 23, 24].

**Fig. 1:**
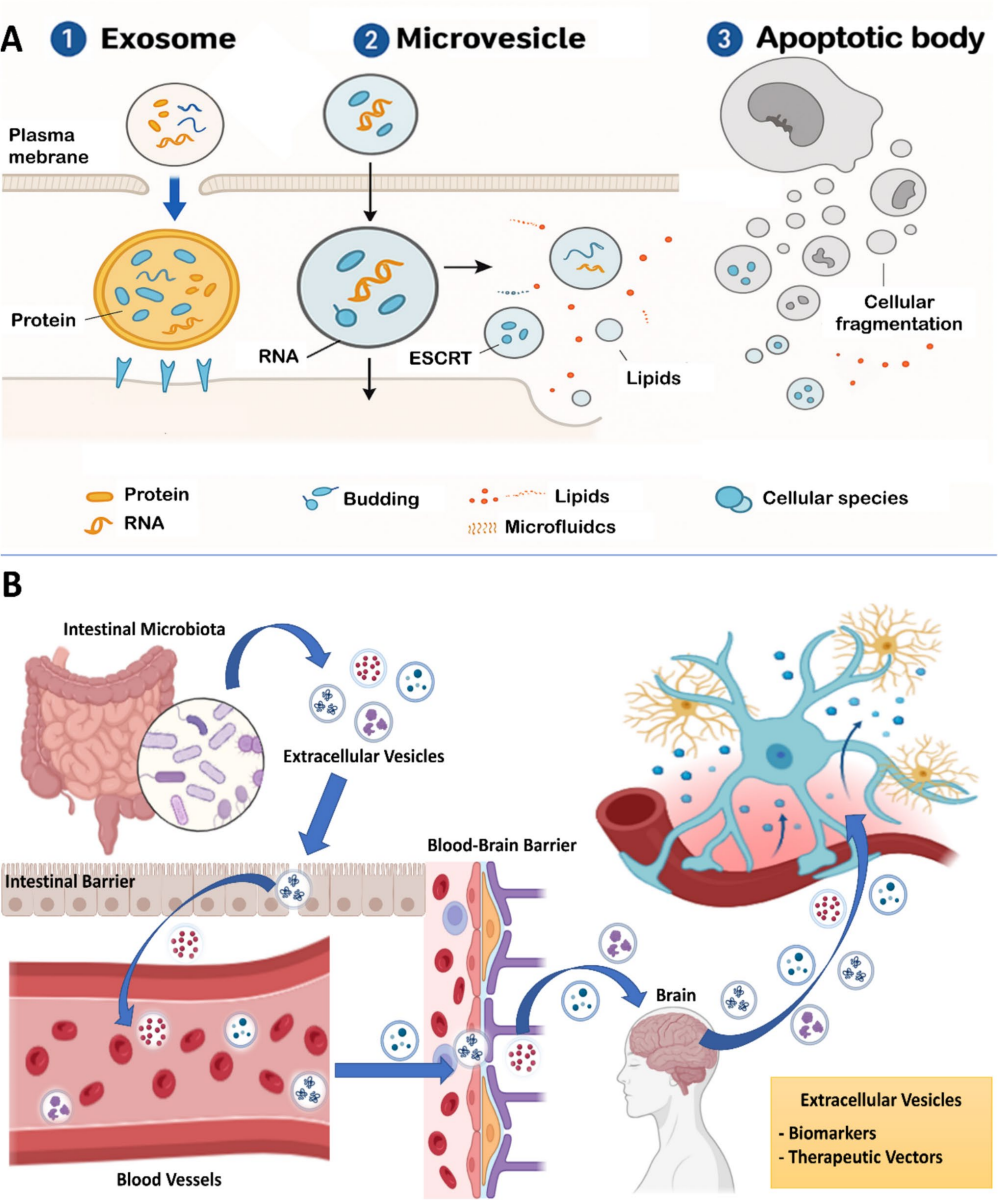
Cellular extracellular vesicle (EV) biogenesis and microbiota–brain integration mediated by microbial EVs: pathways, barriers, and clinical applications **A** illustrates the biogenesis of extracellular vesicles (EVs), which occurs through three main pathways: (1) generation of exosomes within endosomal multivesicular bodies, followed by fusion with the plasma membrane; (2) direct budding of the plasma membrane to form microvesicles; and (3) cellular fragmentation during apoptosis, resulting in apoptotic bodies. Each pathway involves distinct cargo-loading mechanisms governed by vesicular trafficking machinery, ESCRT complexes, and specific lipid components, thereby conferring partially unique biochemical signatures to EV subpopulations. **B** presents a schematic representation of extracellular vesicles mediating communication along the gut–brain axis. The intestinal microbiota releases EVs into the lumen, which cross the intestinal barrier and enter the bloodstream, where they circulate together with host-derived vesicles. After reaching the cerebral microvasculature, subsets of EVs traverse the blood–brain barrier and interact with neural and glial cells, modulating brain function. Owing to their ability to carry both microbial and host-derived molecular cargo, these EVs can be exploited as minimally invasive biomarkers and as therapeutic vectors in gut–brain axis disorders

EV biogenesis (Fig. 1A) occurs through at least three main pathways: generation of exosomes within multivesicular endosomal compartments followed by fusion with the plasma membrane; direct budding of the plasma membrane to form microvesicles; and cell fragmentation into apoptotic bodies during apoptosis [8, 14, 17, 25]. Each pathway involves distinct loading mechanisms, regulated by vesicular trafficking machinery, endosomal sorting complexes required for transport (ESCRT) and specific lipid components, which confer partially unique biochemical signatures to the different EV subpopulations [17, 25–27]. Table 1 summarizes representative markers and studies used to characterize these vesicles in different contexts.

**Table 1:** Characterization of EVs and their main markers

Type of Vesicle	Characterization	Main Markers	Description/Utility	Reference
**Exosomes**	50–150 nm; Endosomal origin	CD63, CD81 (tetraspanins)	Typical exosome membrane markers; used in immunocapture and characterization	Welsh et al. (2024) [6]
		TSG101	Involved in ESCRT-mediated biogenesis; cytosolic marker of exosomes	van Niel et al. (2018) [8]
**Microvesicles**	100–1000 nm; Origin in plasma membrane budding	ARF6	GTPase that regulates direct budding from the plasma membrane	Chuo et al. (2018) [26]
		VAMP3	SNARE protein associated with microvesicle release	Bahmani & Ullah (2022) [29]
		Integrins (e.g., αvβ3, β1)	Involved in adhesion and signaling; present in specific microvesicle subtypes	Agrahari et al (2019) [36]
**Apoptotic bodies**	Origin of cell apoptosis	Phosphatidylserine (PS)	Externalized during apoptosis; detected by Annexin V	Caruso & Poon (2018) [28]
		Histones (H2A, H2B, H3)	Present in nuclear fragments contained in apoptotic bodies	Sheta et al. (2023) [113]
		Cleaved caspase-3	Indicator of apoptotic pathway activation	Welsh et al. (2024) [6]
		Fragmented DNA (TUNEL⁺)	Nucleosomal fragments are released during apoptosis	Yáñez‐Mó et al. (2015) [35]

The heterogeneity of EVs is evidenced not only by their size variation—from 30–150 nm in exosomes to 100–1000 nm in microvesicles and up to several micrometers in apoptotic bodies—but also by differences in protein composition, lipid profile and RNA content, reflecting the identity and functional state of their cells of origin [17, 27–29]. Advances in high-resolution flow cytometry, single-particle imaging and multi-omics approaches have enabled more precise characterization of this diversity, including the identification of vesicle subsets enriched in specific disease-associated cargo [9, 25, 30].

In this mini-review, we use the term brain-derived extracellular vesicles (BDEVs) to refer to vesicles released by neurons, glial and cerebrovascular cells that can cross the BBB and reach peripheral biofluids, and gut bacterial extracellular vesicles (GBEVs) to denote membrane vesicles produced by intestinal microbiota (Fig. 1B) [14, 19, 31–34]. BDEVs can carry proteins associated with neurodegeneration (such as amyloid-β and tau), synaptic proteins and markers of inflammatory stress, thereby serving as non-invasive windows for the early detection and monitoring of CNS disorders, including AD and Parkinson’s disease [12, 15, 19, 33]. In contrast, GBEVs transport lipopolysaccharides, cell-wall components and metabolites, including short-chain fatty acids and tryptophan derivatives, that influence intestinal permeability, immunometabolic signaling and redox homeostasis at the gut–brain interface, shaping both local and systemic responses [14, 16, 23, 34, 35].

Beyond their physiological roles, EVs have shown great promise as diagnostic and therapeutic tools in precision medicine. Their cargo composition mirrors the phenotype of their cell of origin, and they can be engineered to deliver therapeutic molecules with relative specificity and low immunogenicity [22, 27, 29, 36]. Innovative isolation and detection methods—including microfluidic devices, functional magnetic nanoparticle systems and label-free spectroscopic platforms—are improving EV purity and yield, thus enabling more robust application of EVs in advanced molecular diagnostics and targeted therapies for gut–brain axis disorders [18, 22, 36–40].

Understanding the complexity and versatility of EVs in terms of biogenesis, cargo and functional effects is therefore essential for advancing their diagnostic and therapeutic exploitation in gut–brain axis disorders and for designing rational trial strategies based on individualized molecular signatures.

### Gut–Brain Axis Disorders and Extracellular Vesicles

Gut–brain axis (GBA) disorders result from dysfunction in the bidirectional communication between the gastrointestinal tract and the CNS, involving alterations in the gut microbiota, increased epithelial permeability and imbalances in immunometabolic signaling [1–3]. These factors converge to trigger a wide range of gastrointestinal and neurobehavioral manifestations, such as abdominal pain, anxiety, depression and cognitive impairment. EVs, particularly BDEVs and GBEVs, are increasingly recognized as pivotal mediators and potential biomarkers across this spectrum.

### Irritable Bowel Syndrome

Irritable bowel syndrome is one of the most extensively studied models of disrupted gut–brain communication. In IBS, visceral hypersensitivity is commonly associated with intestinal dysbiosis and translocation of bacterial endotoxins, which activate enteric nociceptors and trigger low-grade inflammatory responses both in the myenteric plexus and in CNS regions involved in pain perception and mood regulation [4, 5, 41]. These processes are frequently accompanied by symptoms such as anxiety and depression, underscoring the close relationship between gut health and neuropsychological balance.

At the molecular level, GBEVs play a central role in transporting lipopolysaccharides, cell-wall components and bioactive metabolites, including short-chain fatty acids and tryptophan derivatives [14, 23, 34]. These cargos can modulate expression of epithelial tight-junction proteins (e.g., occludin and ZO-1) and promote the release of inflammatory cytokines such as IL-6 and TNF-α, thereby exacerbating intestinal permeability and perpetuating a cycle of cross-inflammation between the gut and the brain [14, 16, 35]. Parallel changes in circulating EV cargo—including specific miRNAs and long non-coding RNAs—have been associated with disease activity and treatment response in inflammatory bowel disease and related conditions, suggesting that EV signatures may serve as minimally invasive biomarkers in gastroenterology [1, 2, 4].

Simultaneously, BDEVs released by neurons and glial cells can carry microRNAs, inflammatory proteins and signaling peptides that sensitize microglia and alter neurotransmission patterns. These vesicles may contribute to the exacerbation of visceral pain, hypervigilance and cognitive deficits observed in individuals with chronic gastrointestinal dysfunctions [10, 12, 42, 43]. EV-mediated crosstalk between peripheral immune activation and central circuits may therefore represent a mechanistic substrate for the high comorbidity between IBS, IBD and affective disorders.

#### Neurodegenerative Disorders Within the GBA Framework

Beyond classical gastroenterological conditions, growing evidence indicates that the GBA is implicated in the pathophysiology of neurodegenerative diseases such as AD and Parkinson’s disease. Intestinal dysbiosis and increased abundance of bacterial EVs in systemic circulation can promote exacerbated peripheral inflammation and facilitate translocation of microbial components across a compromised BBB [11, 13, 21, 23, 44]. This process may enhance aberrant deposition of proteins such as tau and α-synuclein in vulnerable brain regions, thereby accelerating neurodegeneration [11, 13, 44, 45].

Concurrently, BDEVs isolated from plasma and cerebrospinal fluid in patients with AD and Parkinson’s disease carry disease-relevant cargo, including misfolded proteins, synaptic markers and inflammatory mediators [5, 10, 12, 15, 21, 23, 46]. These vesicles are being actively explored as liquid biopsy tools to detect early pathological changes, stratify patients and monitor progression or therapeutic response. In the context of GBA disorders, the interplay between microbial EVs, systemic inflammation and BDEVs provides a mechanistic framework linking gut dysbiosis to CNS vulnerability.

Notably, not all microbial EVs exert deleterious effects. EVs derived from commensal bacteria such as *Akkermansia muciniphila* may induce anti-inflammatory responses, preserve intestinal barrier integrity and attenuate neuroinflammation in experimental models, highlighting their potential as therapeutic agents or biomarkers of a protective microbiome configuration [47, 48].

#### Psychiatric and Stress-Related Disorders

Alterations in gut microbiota composition and function have been increasingly associated with depression, anxiety and autism spectrum disorders, among others. Microbiota-derived EVs can reach the circulation and interact with immune cells, endothelial cells and possibly neural structures, shaping systemic cytokine profiles and stress-axis regulation [49–56]. Preclinical studies suggest that specific microbial EV signatures may correlate with behavioral phenotypes, while EV-targeted interventions can modulate anxiety-like and depressive-like behaviors [56–64].

In parallel, stress and psychiatric comorbidities frequently observed in IBS and IBD are accompanied by changes in EV cargo derived from both immune and neuronal sources. BDEVs enriched in synaptic, neurotrophic or inflammatory markers may report on maladaptive plasticity within limbic and prefrontal circuits [23, 58, 65–68]. Although clinical evidence is still limited, these observations support the concept that EVs constitute a mechanistic and diagnostic bridge between gut dysbiosis, immune activation and psychiatric symptomatology.

Taken together, EVs emerge as central players in GBA disorders, linking microbial ecology, epithelial and immune status, and CNS function. This has stimulated the development of EV-based biomarkers for diagnosis, prognosis and treatment monitoring, as summarized in Table 2. Proteomic and miRNA signatures in circulating EVs are being explored to predict IBS severity and therapeutic response, while BDEVs and GBEVs are under investigation as readouts of neuroinflammation and cognitive decline in neurodegenerative diseases [15, 44, 69–76].

**Table 2:** Key studies on the gut–brain axis and EVs

Authors (Year)	Experimental Model	Main Findings	Study Potential Clinical Utility
Koloski et al. (2012) [4]	Population cohort (12 years)	Demonstrated gut–brain axis bidirectionality and gut → brain dominance in IBS	Establishes bidirectionality of the GBA and supports clinical rationale for targeting gut and brain simultaneously in IBS management
Carabotti et al. (2015) [1]	Narrative review	Defined neural, endocrine, immune, and metabolic pathways of the gut-brain axis	Provides conceptual framework for multimodal diagnostic approaches integrating neural, endocrine, immune and metabolic markers
Yáñez‐Mó et al. (2015) [35]	Conceptual and experimental review	Described physiological functions, composition, and mechanisms of EVs	Summarizes EV biology and functions, informing selection of candidate EV markers and minimal characterization criteria
Théry et al. (2018) [37]	Update of MISEV guidelines	Established minimum criteria for EV studies (MISEV2018)	Defines MISEV2018 standards, which underpin reproducible EV-based biomarker discovery and future clinical validation
Aharon et al. (2020) [5]	Alzheimer's patients	Circulating EVs as biomarkers of clinical progression in Alzheimer's disease	Demonstrates that circulating EVs can track clinical progression in AD, supporting use of BDEVs as prognostic biomarkers
Villard et al. (2021) [14]	Metabolic syndrome model in rats	GBEVs modulate insulin resistance and systemic inflammation	Shows that GBEVs modulate metabolic and inflammatory pathways in metabolic syndrome, suggesting EV-based markers for metabolic risk in GBA disorders
Cuesta et al. (2021) [23]	In vitro and in vivo study in mice	Microbiota-derived EVs regulate intestinal permeability and immunity	Indicates that microbiota-derived EVs regulate intestinal permeability and immunity, providing mechanistic basis for stool- or plasma-EV biomarkers
Zhao et al. (2025) [47]	Murine model of neuroinflammation	EVs from *Akkermansia muciniphila* preserve the intestinal barrier and reduce neuroinflammation	Demonstrates that *A. muciniphila* EVs preserve intestinal barrier and reduce neuroinflammation, highlighting therapeutic and biomarker potential of beneficial microbial EVs
Welsh et al. (2024) [6]	MISEV2023 (update and standardization)	Methodological harmonization in EVs for clinical applications	MISEV2023 update, offering advanced guidelines for clinical-grade EV studies and translational trial design
Jung et al. (2024) [16]	Gut-brain chip with human microenvironment	GBEVs modulate mitochondria and protein aggregation associated with Alzheimer's	Uses a human gut–brain chip to link GBEVs, mitochondria and protein aggregation, illustrating how EV readouts can refine mechanistic and pharmacological studies

This table combines the key studies on the gut–brain axis (GBA) and extracellular vesicles (EVs), encompassing both experimental research and conceptual reviews, as well as fundamental methodological guidelines in the field.

### Diagnostic Tools and Analytical Platforms for EVs In Gut–Brain Axis Disorders

EVs have emerged as promising diagnostic tools due to their ubiquitous presence in bodily fluids and their ability to reflect tissue- and cell type–specific molecular signatures, including membrane proteins, lipids, mRNAs, miRNAs, and metabolites [6, 7, 24, 35, 77, 78]. Their relative stability in complex biological matrices and capacity to cross physiological barriers further enhance their potential for non-invasive diagnostics and precision medicine applications.

### Physical Isolation Approaches

Conventional isolation techniques such as differential ultracentrifugation, density gradients, tangential flow filtration and polymer-based precipitation remain widely used in EV research and have been applied to GBA-related studies (Table 3). These methods are scalable and relatively accessible but present important limitations: lengthy processing times, operator dependency and co-isolation of contaminants such as soluble proteins and lipoproteins, which compromise marker specificity and assay reproducibility [14, 18, 37, 79–81].

**Table 3:** Comparison between extracellular vesicle isolation and characterization techniques

Technique/Platform	Analytical principle	Typical sample matrix	Main advantages	Main limitations	Clinical readiness*	Typical readouts/biomarkers	Notes on MISEV2023 compliance	Exemplary refs
Differential ultracentrifugation (DUC)	Physical (size/density; high g-force pelleting)	Plasma/serum, CSF, stool supernatant, cell CM	Widely available; scalable volumes; historical comparability	Co-pellets proteins/lipoproteins; operator-dependent; time-consuming	Exploratory clinical	Bulk EV yield; downstream WB/omics	Must report rotor/k-factor, times, speeds; orthogonal characterization recommended	[18, 37, 80]
Density-gradient ultracentrifugation (DGUC)	Physical (isopycnic separation)	Plasma/serum, CSF, CM	Higher purity vs DUC; separates by buoyant density	Low throughput; long runs; specialized reagents	Exploratory clinical	Enriched small EV fractions; proteo/miRNomes	Document gradient medium; verify density + markers	[18, 37]
Tangential flow filtration (TFF)	Physical (membrane, cross-flow)	Large plasma/serum volumes, CM, stool extracts	Scalable, gentle; integrates with SEC	Membrane fouling; device cost	Exploratory clinical	Concentrated EV suspensions	Report membrane cutoff/flux; pair with orthogonal purity checks	[80]
Size-exclusion chromatography (SEC)	Physical (size-based elution)	Plasma/serum, CSF, urine, stool	Good purity; preserves EV integrity; reproducible	Dilution of sample; fraction collection required	Exploratory → Near-POC (when standardized kits)	Cleaner proteo/miRNomes; NTA counts	Report column specs/fractions; assess lipoprotein carry-over	[18, 82]
Polymer precipitation (e.g., PEG)	Physical–chemical (volume-exclusion)	Plasma/serum, urine, CM	Simple; low equipment needs	High co-precipitation (proteins, HDL/LDL); poor purity	Preclinical	Screening proteomics/miRNA (discovery only)	Not preferred for diagnostics; requires extra clean-up	[18]
Immunocapture (tetraspanins CD9/CD63/CD81)	Affinity (antibody capture)	Plasma/serum, CSF, urine, stool	Subpopulation enrichment; amenable to automation	Epitope masking; variable recovery; cost	Exploratory clinical	Vesicle counts; surface proteome; miRNA cargo	Specify antibody clones, elution, negative controls	[36, 39]
Cell-type EV enrichment (e.g., “neuronal” L1CAM)	Affinity (cell-marker targeting)	Plasma/serum, CSF	Putative cell-of-origin signal; hypothesis-driven panels	**Marker specificity controversy (L1CAM)**; soluble protein carry-over	Exploratory clinical	CNS-linked proteins (Aβ/tau), synaptic markers, miRNAs	Include bead-only controls; verify vesicular nature; orthogonal validation	[35, 37, 39, 44]
Magnetic bead/nanoparticle capture	Affinity (magnetic ligands/antibodies/aptamers)	Plasma/serum, CSF, urine	Fast workflows; integrates with chips/readers	Ligand stability; non-specific binding	Exploratory → Near-POC	On-bead ELISA, electrochemical readouts	Detail ligand chemistry; spike-in/process controls	[38, 39]
Microfluidic immunocapture chips	Microfluidic (on-chip capture/processing)	Low-volume plasma/serum/CSF, stool	Minimal sample; multiplex; short TAT	Device cost; fabrication variability	Near-POC (pilot)	Multi-marker signatures; integrated lysis-omics	Report flow rates, channel geometry; replicate runs	[38, 39]
Interferometric imaging (digital detection)	Label-free optics (interference of single EVs)	Plasma/serum, CSF	Single-particle sensitivity; counting + sizing	Instrument availability; calibration	Exploratory clinical	Particle concentration/size distributions	Calibrate with standards; link counts to cargo	[85]
Impedance/electrochemical microscopy	Label-free electrochemical	Plasma/serum, CSF, urine	Real-time, reagent-light; portable potential	Surface fouling; matrix effects	Near-POC (prototype)	EV counts; antigen-specific signals (with ligands)	Electrode cleaning/logs; external QC	[87]
SPR/optofluidic plasmonics	Label-free optics (SPR, interferometry)	Plasma/serum, CSF	High sensitivity; kinetics; multiplexing	Instrumentation; surface chemistry optimization	Exploratory → Near-POC	Binding kinetics; surface protein panels	Document chip chemistry; use blanks/negatives	[31]
Spectroscopic fingerprints (Raman/FTIR)	Label-free spectroscopy (bulk/ensemble)	Plasma/serum, urine, stool	Rapid phenotyping; low reagents	Deconvolution; needs robust ML	Preclinical → Exploratory	Global “EV signature” for classification	Provide ML pipeline; external validation sets	[40]
NTA (Nanoparticle Tracking Analysis)	Characterization (Brownian motion)	Any EV prep	Size distribution; particle counts	Refractive-index bias; operator variance	Supporting (all stages)	QC metric; normalization	Report camera settings/thresholds; repeatability	[114]
High-sensitivity flow cytometry	Characterization (light scatter/fluorescence)	Any EV prep (esp. plasma)	Phenotyping by surface markers; counting	Small-particle detection limits; swarm	Supporting (all stages)	Single-EV immunophenotype	Detail triggering, calibration beads, swarming controls	[115]
Proteomics (DDA/DIA) & miRNA-seq	Downstream analytics (omics)	Plasma/serum, CSF, stool	Deep biomarker discovery; pathway insights	Batch effects; input requirements	Exploratory → Clinical (panel-based)	Diagnostic panels (protein/miRNA)	Include spike-ins; cross-platform validation; FAIR data	[15, 88, 89]
Paper-based & smartphone-read POC assays	Point-of-care (lateral-flow/aptasensor)	Plasma/serum, saliva, urine	Portable; low-cost; rapid	Currently disease-specific, few EV panels	Prototype → Near-POC	Colorimetric/electrochemical EV targets	Document LoD, reproducibility, external QC	[83, 84]

Clinical readiness (definitions used in the review): Preclinical (research/laboratory use); Exploratory clinical (early human cohorts, limited validation); Near-POC (prototypes/clinical studies with potential for bedside or routine use). Abbreviations: CSF, cerebrospinal fluid; CM, conditioned medium; and POC, platforms for point-of-care.

Size-exclusion chromatography offers higher purity and preserves vesicle integrity but may be less efficient for large volumes and requires careful standardization [82]. In the context of clinical translation, physical methods are often used in combination (e.g., tangential flow filtration followed by size-exclusion chromatography) to balance yield and purity, yet many GBA-EV studies still provide incomplete methodological reporting and partial characterization, falling short of MISEV2018/2023 recommendations [6, 7, 37].

#### Affinity-Based Enrichment of Disease-Relevant EVs

Affinity-based strategies—including immunocapture using antibodies against tetraspanins (CD63, CD81, CD9) or cell-specific markers—enable enrichment of EV subpopulations from plasma, cerebrospinal fluid or stool [22, 36, 39]. Magnetic nanoparticles functionalized with antibodies targeting presumed neuronal markers (e.g., L1CAM) or other cell-type–associated surface proteins have been used to isolate BDEVs in AD, multiple sclerosis and cancer [21, 39, 46]. These approaches can achieve high specificity and are amenable to automation but raise concerns regarding marker specificity, potential co-isolation of soluble proteins and cost.

The controversy surrounding L1CAM as a neuronal EV marker illustrates how incomplete knowledge of EV surface landscapes can bias diagnostic interpretation and highlights the need for rigorous negative controls and orthogonal validation [37, 39]. For microbial EVs, affinity-based capture using bacterial surface antigens is conceptually attractive but remains technically challenging due to the diversity of microbiota-derived vesicles and incomplete annotation of their surface markers [14, 31, 32, 34].

#### Microfluidic and Label-Free Platforms for Point-of-Care Applications

Microfluidic devices have gained prominence by integrating multiple workflow steps—from selective capture and washing to on-chip detection—into miniaturized and automated platforms [38]. These systems operate with minimal sample volumes and can be functionalized with antibodies, aptamers or other ligands to capture EVs from plasma, urine or cerebrospinal fluid with high specificity. In addition to accelerating processing, microfluidic devices enable multiplexed, high-throughput analyses, making them attractive for large-scale screening and eventually POC applications [38, 83, 84].

Label-free optofluidic platforms based on electrical impedance, interferometry or surface plasmon resonance can quantify EVs in real time without fluorescent labeling, reducing assay complexity and cost [31, 40, 85–87]. Combined with multi-omics analysis of EV cargo (proteomics, miRNA profiling, metabolomics) and machine-learning approaches, these technologies have identified disease-specific EV signatures in cardiovascular, neurodevelopmental and neurodegenerative conditions [88, 89]. Their adaptation to IBS, IBD and neurogastrointestinal disorders is an active area of research and may support personalized diagnostics by integrating EV-based biomarkers with clinical and imaging data.

#### Three Niches for EV-Based Diagnostics in Gut–Brain Axis Disorders

**Fig. 2:**
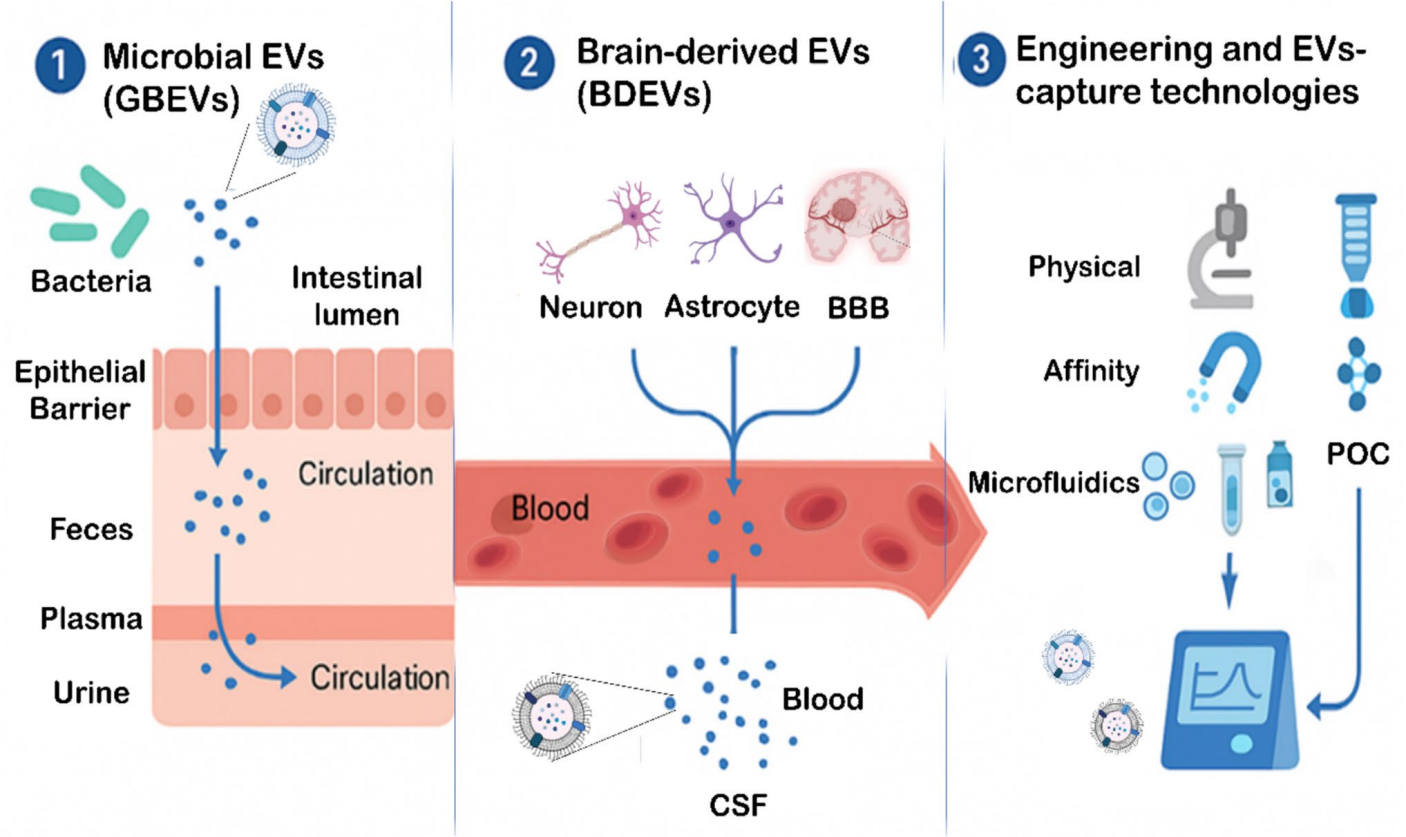
Conceptual pipeline for EV-based diagnostics in gut–brain axis disorders The schematic illustrates three interconnected diagnostic niches along a translational pipeline. (1) Microbial EVs (GBEVs) are released into the intestinal lumen and cross the epithelial barrier, entering the circulation where they can be profiled in stool, plasma or urine as systemic markers of dysbiosis, barrier integrity and low-grade inflammation. (2) Brain-derived EVs (BDEVs) originating from neurons, glial and cerebrovascular cells cross the blood–brain barrier and reach blood and cerebrospinal fluid, providing a liquid biopsy of central nervous system (CNS) pathology linked to gut–brain axis dysfunction. (3) Engineered and technologically captured EVs are isolated using physical, affinity-based or microfluidic approaches and analyzed for their protein, lipid and RNA cargo with advanced platforms, enabling multiplexed and point-of-care readouts. Together, these niches have the potential to support early diagnosis, disease stratification and treatment monitoring across irritable bowel syndrome, inflammatory bowel disease, neurodegenerative and psychiatric disorders. Caption: Blood–brain barrier (BBB), Cerebrospinal fluid (CSF), and Platforms for point-of-care (POC). Abbreviations: EVs, extracellular vesicles; GBEVs, gut bacterial EVs; BDEVs, brain-derived EVs; BBB, blood–brain barrier; CSF, cerebrospinal fluid

From a translational perspective, current evidence on EVs in GBA disorders can be organized as a conceptual pipeline composed of three complementary diagnostic niches (Fig. 2) that move from systemic screening to CNS-focused readouts and, ultimately, point-of-care (POC) implementation:

1. Microbial EVs as systemic sentinels (screening and risk stratification): In the first tier of the pipeline, GBEVs and other microbiota-derived EVs detected in stool, plasma or urine report on dysbiosis, epithelial barrier function and low-grade inflammation. Experimental models and emerging human data indicate that their protein and RNA cargos correlate with mitochondrial dysfunction, neuroinflammatory signaling and behavioral phenotypes relevant to IBS, metabolic syndrome and AD [14, 16, 23, 31, 32, 34, 35, 47, 48]. Profiling GBEVs can therefore serve as an initial screening step to identify at-risk individuals, support early risk stratification and monitor interventions aimed at restoring microbiota–host homeostasis.
2. Brain-derived EVs as CNS liquid biopsies (central validation and staging): A second tier of the pipeline focuses on BDEVs isolated from blood and cerebrospinal fluid, which carry misfolded proteins, synaptic markers and neuroinflammatory mediators that reflect ongoing CNS pathology [5, 10, 12, 15, 19, 21, 23, 33, 46, 90]. In the context of GBA disorders, BDEVs provide a mechanistic link between systemic alterations (inflammation, dysbiosis) and central changes. Longitudinal studies suggest that BDEV cargo could anticipate cognitive decline, track disease progression and validate the central impact of gut-directed or immunomodulatory therapies identified in the first screening tier.
3. Engineered and technologically captured EVs for POC testing (clinical deployment): In the final tier, microfluidic, affinity-based and nanoplasmonic platforms can enrich disease-relevant EV subpopulations from small sample volumes and generate multiplexed readouts compatible with point-of-care formats [31, 38, 39, 83, 84, 87]. In GBA disorders, these technologies could be used to rapidly detect EV biomarker panels associated with IBS severity, IBD activity or early neurodegeneration in patients with chronic gut inflammation, delivering actionable information at the bedside or in outpatient settings.

Taken together, this tripartite pipeline illustrates how EVs from distinct sources—microbial, intestinal and brain—can be integrated in a sequential and complementary fashion, from systemic screening to CNS validation and bedside deployment, to inform diagnosis, staging and longitudinal management of neurogastrointestinal diseases.

#### Translational Hurdles and Methodological Challengesin Gut–Brain Axis Disorders

Despite encouraging advances, several hurdles still limit the clinical implementation of EV-based diagnostics specifically in gut–brain axis (GBA) disorders.

First, pre-analytical variability—including diet, circadian rhythm, stool consistency, sampling frequency and storage conditions—strongly influences EV yield and cargo profiles, particularly for stool- and plasma-derived EVs that are central to GBA research. Many studies in patients with gut–brain axis disorders do not yet fully report or control these parameters, making it difficult to disentangle disease-related signals from background variability [6, 15, 44, 69–74].

Second, isolation and characterization methods differ in purity and recovery, and a substantial proportion of published work in the GBA field only partially complies with MISEV2018/2023 recommendations regarding documentation of protocols, use of appropriate controls and multiparametric characterization [6, 7, 14, 18, 37]. This reduces comparability across studies of gut–brain axis disorders and complicates meta-analyses aimed at validating candidate EV biomarkers that link intestinal and central nervous system (CNS) pathology.

Third, the specificity of surface markers used to enrich cell-type– or compartment-specific EVs (e.g., “neuronal”, “microglial”, “intestinal epithelial” or “bacterial” vesicles) remains incompletely defined in the context of GBA disorders. Marker controversies, as exemplified by L1CAM, illustrate how soluble proteins, non-vesicular particles and off-target binding can confound interpretation of EV-based biomarkers purported to reflect gut or brain compartments, underscoring the need for orthogonal validation strategies and careful negative controls in gut–brain axis studies [91–98].

Fourth, inter-individual variability—driven by genetics, diet, microbiota composition, comorbidities, medications and lifestyle—is particularly pronounced in gut–brain axis disorders and challenges the definition of robust diagnostic thresholds [99–105]. This scenario calls for large, well-phenotyped cohorts and longitudinal sampling that capture both gastrointestinal and neuropsychiatric dimensions of GBA phenotypes.

Finally, regulatory agencies will demand robust evidence that EV-based assays add measurable clinical value over existing diagnostic and monitoring tools used in gut–brain axis disorders, such as endoscopy, imaging, standard inflammatory markers and neuropsychological assessments [6, 106–111]. In addition, harmonized protocols, external quality assessment, scalability and cost-effectiveness will be required before EV-based tests can be adopted for routine management of GBA disorders [6, 106, 106, 107, 112]. Addressing these issues will depend on close alignment with consensus guidelines, interdisciplinary collaboration between gastroenterology, neurology, psychiatry and laboratory medicine, and early engagement with regulatory bodies to tailor validation pathways to the specificities of gut–brain axis conditions.

## Conclusion

EVs are emerging as promising diagnostic tools in the context of gut–brain axis disorders due to their ability to reflect specific molecular signatures, traverse biological barriers and integrate signals from the microbiota, intestinal epithelium, immune system, and CNS. Ongoing advances in isolation and characterization technologies are progressively enabling increasingly precise identification of vesicle subtypes and cargos, supporting their use in non-invasive diagnostics and personalized medicine.

By framing current evidence into three diagnostic niches—microbial EVs as systemic markers, BDEVs as CNS liquid biopsies and engineered/technologically captured EVs for POC testing—this mini-review highlights how different EV sources can be strategically combined to improve early detection, risk stratification and treatment monitoring in IBS, IBD, neurodegenerative and psychiatric disorders.

Nevertheless, significant challenges remain regarding methodological standardization, marker specificity, pre-analytical and inter-individual variability, and regulatory approval. Future studies should prioritize compliance with MISEV2023 guidelines, use of multi-omics approaches in well-characterized cohorts and rigorous validation of candidate biomarkers across independent populations. As these hurdles are overcome, EV-based diagnostics are poised to become central components of precision medicine strategies targeting neurogastrointestinal disorders.

## Data Availability

No datasets were generated or analysed during the current study.
